# The impact of the peer effect on adolescent drinking behavior: instrumental-variable evidence from China

**DOI:** 10.3389/fpsyt.2023.1306220

**Published:** 2023-12-21

**Authors:** Meng Liu, Wen-Qing Zhao, Qi-Ran Zhao, Yu Wang, Shun-Guo Li

**Affiliations:** ^1^Institute of Millet Crops, Hebei Academy of Agriculture and Forestry Sciences, Shijiazhuang, Hebei, China; ^2^College of Economics and Management, China Agricultural University, Beijing, China

**Keywords:** peer effect, adolescent addiction, drinking behavior, instrumental variable, China

## Abstract

**Background:**

Governments around the world have taken measures to limit adolescent drinking, however, rates are still alarmingly high. However, most of these measures ignore the peer effect of drinking among adolescents. Previous studies have not sufficiently considered the reciprocal relationship between adolescent alcohol consumption and peer alcohol consumption, which may lead to an overestimation of the peer effect and mask underlying issues. Good instrumental variables are powerful but rare tools to address these issues.

**Objective:**

This paper aims to correctly estimate the peer effect of drinking on adolescent drinking behavior in China.

**Methods:**

Owing to the detailed information of household background in the dataset of our survey, we were able to use the drinking behaviors of peers’ fathers and their beliefs about the health risks of alcohol as instrumental variables, which are more powerful than school-average instrumental variables. We collected data from the 2017 Health and Nutrition Panel survey, which surveyed 10,772 primary school students from 59 urban migrant and 60 rural public schools.

**Results:**

The instrumental variable method estimation revealed that peer drinking significantly influences adolescent drinking behavior, with adolescents who have peers who drink alcohol being 10.5% points (2 stage least square, i.e., 2SLS, full sample estimation) more likely to engage in drinking compared to those without such peers. Furthermore, the effect differs significantly between migrant and rural adolescents.

**Conclusion:**

The study found that parental care plays a significant role in the degree of peer effect, with the absence of parental care being a key factor in the presence of the peer effect.

## 1 Introduction

Adolescent drinking is on the rise (1). In a study by Hibell et al. (2), 15–16-year-old students in 35 European countries reported that approximately 90% (range: 66–95%) of youth have tried alcoholic beverages at least once. Recent data from the World Health Organization has shown that 41.2% of 15–19-year-olds drink alcohol in China (3). According to a meta study, among Chinese adolescents, the prevalence rates for lifetime drinking, past month drinking, and binge drinking are 51, 24, and 9%, respectively (4).

Underage drinking can have profound negative consequences for underage drinkers, as well as their families, their communities, and society as a whole (5). According to a 2014 estimate by the WHO, 5.1% of the global burden of disease and injury can be attributed to alcohol consumption (6). Alcohol consumption can cause more than 200 illnesses and injuries (3), and can increase risk for type 2 diabetes, coronary artery disease, arrhythmias, and stroke among adolescents who consume alcohol early in life (7). Additionally, studies have shown that partaking in harmful drinking behavior at a young age increases one’s risk of serious mental illness and excessive drinking in adulthood (8). Problematic drinking behavior can also lead to a range of social problems such as violence, crime, and suicide (9, 10). Moreover, data from the 2004 WHO Global Burden of Disease Study indicates that alcohol consumption itself is a major risk factor for decreasing disability-adjusted life years among people aged 10–24 years (11).

Given the risks, governments around the world have taken various measures to limit teenage drinking. Rates of adolescent alcohol have varied across country contexts due to the different cultural practices, laws, and policies. Countries, such as the United States, that emphasize zero-tolerance policies seek to encourage adolescent abstinence from alcohol and drug use (12). In order to do so, the United States has issued laws and regulations that restrict teenage drinking behavior (5). Similarly, China established Article 67 of the Law on Protection of Minors, which stipulates that those who sell tobacco or alcohol to minors, or fail to set up conspicuous signs not to sell tobacco or alcohol to minors, shall be ordered to make corrections by the competent authorities and given administrative punishment. It indicates that although selling tabaco and alcohol to minors is still non-criminal, those who violates this administrative regulatory will face with penalties or sanctions range from fines and warnings to the suspension of certain privileges. Experts and scholars recommend intervention in adolescent drinking behavior from the aspects of retail availability (13, 14), social availability (15), pricing (16–18), and drinking and driving (19–21). Nevertheless, underage drinking rates remain alarmingly high, particularly among youth aged 18–20 years, causing preventable health and safety consequences (5). A meta-analysis of drinking behavior among Chinese adolescents, showed that 15.3–44.7% of secondary school students drank alcohol (22).

Adolescent drinking behavior has been shown to be related to social and economic factors. Adolescents with downward socioeconomic mobility during childhood are more likely to drink alcohol (23), and studies have shown that cultural orientation influences adolescent drinking behavior in developing countries (24). Among the many socioeconomic factors that influence adolescent drinking, family is considered to be an important social factors affecting the occurrence and transformation of underage drinking problems (25). First, compared to living with parents, living with other family members is associated with a wide variety of problematic behaviors in adolescents (26). Additionally, Barrett and Turner found family structure was associated with adolescent drinking behaviors (26). Further, a Chilean study found that parental drinking was one of the main causes of teenage drinking (27). In the study of gender heterogeneity, Bobakova et al. found that females appear to be sensitive to parental monitoring in regard to drunkenness, while males are not (28). In China, family structure has been shown to have a similar impact on adolescents’ drinking behaviors, with parents’ drinking behaviors reported as having the greatest impact (29).

Although family also has an important influence on adolescent drinking behavior, adolescents spend less time with their parents and more time with their peers (30). Clark and Lohéac and Evans et al. found that there is a close relationship between the behavior of adolescents and the behavior of their peers (31, 32). Thus, peer drinking is widely viewed to have a significant influence on adolescent alcohol use as well (33). Peer alcohol consumption and expectations of alcohol remained the most important predictors of alcohol problems among Spanish adolescents (31). When controlling for other background characteristics, peer influence was the most significant factor affecting adolescent drinking (32, 34). Further, Chuang et al. found that the neighborhood context constitutes the setting in which peer influences on adolescent behaviors occur (35). Barrett and Turner and Gommans et al. also found that the popularity composition of one’s peer group and the relative difference in popularity between an adolescent and their peers is also associated with adolescent drinking (26, 36). Tyler et al. also found that peer drinking lead to higher levels of alcohol abuse among adolescents aged 14–16 (37). Lee et al. also found that having peers who consume alcohol may increase adolescents’ future drinking behaviors by up to 80% (34).

Peer effect has long been viewed as crucial element to students’ human capital accumulation (38). Despite so, for researchers, a point of view that it may be a potent influence on bad behaviors, specially, the alcohol use, can be date back to Bauman and Ennett’s study in 1996 (39) and even before. There is a great deal of research regarding the peer effect on adolescent drinking, but there are still some shortcomings. Adolescents can and do select peers with similar drinking habits (40). They tend to acquire friends who are similar to them, and they also appear to acquire new friends who are similar to their old friends (41). Previous studies do not pay attention to the endogenous problem of mutual causation between adolescents’ and their peers’ drinking during model regression. In this study, we have raised the basic question of whether the peer-teen drinking correlation is due to selection or causation.

Some relative attempts are cross-lagged panel models (42–44) and survival model (45). However, a better approach that is often used in economic researches is the instrumental variable (IV) method. It does not rely on the assumption that lagged behaviors are not correlated with each other. But, a good IV requires additional information that only affect the peer’s drinking behavior and consequently difficult to be found. Fortunately, with the detailed information of parents, we have the chance to detect a good IV. Specifically, in order to overcome the endogeneity of peer influence on adolescent drinking, we took “whether the peer’s father is drinking” and “whether the peer himself/herself believes that drinking alcohol is unhealthy” as instrumental variables to investigate the peer effect on adolescent drinking. These are more powerful than commonly used school-averaged instrumental variables and the reasons are detailed in the “Validity of the instrumental variables” section. In the context of Chinese culture, parental drinking significantly affects adolescent drinking behavior (46), as there is a correlation between a father’s drinking behavior and the drinking behavior of their child (47). But peer’s father’s drinking would not affect their own drinking behavior. Therefore, peers’ fathers’ drinking is regarded as a good instrumental variable of peer drinking.

## 2 Materials and methods

### 2.1 Type of this study

This study is a cross-sectional observational study that uses instrumental variable methods to estimate the peer effect of drinking on adolescent drinking behavior in China. The study uses data from the 2017 Health and Nutrition Panel survey (HNPS), which covers 10,772 primary school students from four provinces in China. The study uses the drinking behaviors of peers’ fathers and their beliefs about the health risks of alcohol as instrumental variables, which are more powerful and valid than commonly used school-average variables. The study also examines the heterogeneity of the peer effect across different groups of adolescents, such as migrant and rural, left-behind and non-left-behind, and parents’ migrant work.

### 2.2 Data

#### 2.2.1 Sampling

The data in this study were obtained from the 2017 Health and Nutrition Panel survey (HNPS), which originates from a project conducted by College of Economics and Management at the China Agricultural University. The HNPS adopted a stratified sampling method to draw approximately 10,772 students from 238 classes of third- and fourth-years (ages of 8 and 10-years) at 119 primary schools across 4 provinces. We used the data from HNPS for three reasons. First, it is a large-scale primary school education survey for China. Second, the survey collected detailed information about the characteristics of students, as well as their families. Third, the HNPS includes students with rural household registrations in both urban and rural areas. Among them, the students in urban were considered as migrant students, while the students in rural include left-behind children and non-left behind children. With rapid urbanization and industrialization of China, many rural residents have migrated to urban areas for work, increasing the proportion of left-behind children in rural villages. Therefore, we can identify if there is a difference between the types of students in terms of the peer effect.

The HNPS employed a multi-faceted methodology that involved the survey of students, collection of data on alcohol consumption, and the administration of standard mathematics tests and health assessments. The sampling procedure was comprised of three distinct phases. First, during the period from May 15th, 2017 to June 15th, 2017, the HNPS data included students with rural registered residences in Beijing (northern China), Suzhou (eastern China), Henan (central China), and Anhui (central China) provinces. Henan is the province that sends the most migrant workers to Beijing, while Anhui province is the largest source of immigrants to Suzhou and one of the largest sources of immigrants to Shanghai (44). Then, the researchers approached the local bureau of education to obtain a comprehensive list of primary schools in these counties (excluding schools located in the county seat as they were predominantly attended by urban children and were not covered by the nutrition improving program neither), from which six schools were randomly selected per county, resulting in a total of 60 schools. Finally, one class from grade 3 and grade 4 was randomly selected from each school to conduct the surveys and tests. Finally, after dropping those who did not finish all the relative questionnaires (parents’ and students’) and tests, a total of 60 classes (1,931 students) in Beijing, 58 classes (3,239 students) in Suzhou, 60 classes (3,456 students) in Henan, and 60 classes (2,934 students) in Anhui. Overall, there were 5,718 third-graders and 5,842 fourth-graders were included in the sample. Due to a little sort of the children are “isolated,” who has not a friend at school, all samples could not be matched with corresponding peers. Therefore, we only used 10,772 samples who have friend at school.

It is worth noting that the students’ parents are informed and have given written consent to the survey. They were informed that the data was collected only for scientific research and will be analyzed only anonymously. The ethnical approval is provided by China Agricultural University Institutional Review Board. Only researchers who are authorized the College of Economic and Management, China Agricultural University can have access to the data.

#### 2.2.2 Data collection

All HNPS participants were asked to answer a series of questions in order to generate variables to measure individual and family characteristics for each student, as well as to collected personal and family information from students and ask who their close friends are in the same class. We collected information on each student’s gender, age, and number of siblings, as well as whether the student participated in preschool. We also collected information regarding the ages and education levels of the students’ fathers and mothers and whether their father consumed alcohol or smoked tobacco. The durability assets of family were also measured. Students’ drinking statuses were obtained by asking them whether they drank alcohol. The identity of students’ peers was determined by asking them what the name of their best friend was. This was matched with the corresponding peer survey. Throughout the paper, when referring to peers, we are referring to the best friend that each student provided in their answer.

Brunborg et al. found that adolescent alcohol consumption was closely related to disposable income, however, income information is not available from the HNPS because it is filled out by students, not parents (45). Despite this, the HNPS can provide information on the main assets owned by a student’s household. The survey asks students about seven main assets, including a refrigerator, television, microwave oven, induction cooker, air conditioner, washing machine and computer. Based on this information, and using the method proposed by Filmer and Pritchett (47), we conducted a principal component analysis (PCA) to create a variable that measures household durable assets to generate a proxy for household wealth. If a household owned a durable asset, it was recorded as 1, otherwise it was recorded as 0. We applied PCA method on these dummy variables to calculate the scoring factor that captures the “relative household asset condition comparing to the other local households.” The descriptive statistic of this variable is shown in Table 1 (Household assets). Notice that it is the relative value, rather than the absolute value of this variable contains the information we want.

**Table 1:** Variables and summary statistics for the student sample.

Variable	Definition	Obs.	Mean	SD
Drink	Dummy, 1 = drinks, 0 = not,	10,772	0.265	–
Drink_peer_	Dummy, 1 = peer drinks, 0 = not,	10,772	0.272	–
Father drinks	Dummy, 1 = father drinks, 0 = not,	10,772	0.749	–
Aware	Dummy, 1 = drinking harmful to health, 0 = not	10,772	0.698	–
Gender dummy	Dummy, 1 = boy, 0 = girl	10,772	0.520	–
Age	Age measured by month	10,772	126.9	10.74
Preschool	Dummy, 1 = attended preschool, 0 = not	10,772	0.932	–
Number of siblings	The number of siblings	10,772	0.468	0.636
Local dummy	Dummy, 1 = rural, 0 = migrant	10,772	0.567	–
Father’s age	Age of father	10,772	37.55	1.534
Mother’s age	Age of mother	10,772	35.95	1.281
Father’s education	Educational years of father	10,772	9.010	2.660
Mother’s education	Educational years of mother	10,772	8.424	3.301
Household assets	Household durable asset index	10,772	0.000	1.470

Data source: author’s survey.

### 2.3 Sample characteristics

After processing and selecting variables, 10,772 student samples were obtained. The explained variable was Drink (whether the student drinks alcohol), and the core explanatory variable was Drink_peer_ (whether the student’s peer drinks alcohol). The control variables were Gender, Age, Preschool (whether the student attended preschool), Number of siblings, Local dummy (rural or migrant), Father’s age, Mother’s age, Father’s education, Mother’s education, and Household assets.

Table 1 reports the individual and family characteristics of the sample. Overall, the drinking behaviors of students were similar to those of their peers. Most students had a certain understanding of the harm of drinking. In our sample, parents were usually young, low level of education, and generally have only one child.

### 2.4 Statistical relationship of interest

The purpose of this study was to estimate the influence of peers on students’ drinking behaviors. The Ordinary Least Squares (OLS) model estimates are as follows:


(1) $$\mathrm{drin}k_{i}=\beta_{0}+\beta_{1}\mathrm{drin}k_{\mathrm{peer},i}+\beta_{2}M_{i}+\beta_{3}N_{i}+\delta_{i}+\varepsilon_{i}$$

Where, $\mathrm{drin}k_{i}$ refers to whether the student (i) has the behavior of drinking, and $\mathrm{drin}k_{\mathrm{peer},i}$ refers to whether the student’s peer has the behavior of drinking. In addition, $M_{i}$ represents a series of characteristic variables at the individual level (for the student), such as gender, age, whether they attended preschool, and number of siblings. $N_{i}$ represents a series of characteristic variables at the household level, such as parents’ ages, parents’ educational years, and household durable assets. $\delta_{i}$ is included to control for differences between schools. $\varepsilon_{i}$ is the error term.

### 2.5 Validity of the instrumental variables

Given the mutual influence of students and their peers’ drinking behaviors, which means they affect each other, the OLS estimates may be biased. To address this endogeneity issue, this study selected the drinking behavior of students’ peers’ fathers and their perception of the harm of drinking as IVs.

Traditionally, two criteria have been used to assess the quality of instrumental variables (IVs): exogeneity and validity. First, there is a strong correlation between the drinking behaviors of students’ peers’ fathers and the drinking behaviors of the students’ peers. However, peers’ fathers’ drinking behaviors have no influence on the students’ own drinking behaviors. Second, there is a high correlation between peers’ understanding of the harm of drinking and their own drinking behavior. However, peers’ understanding of the harm of drinking has no impact on adolescents’ drinking behaviors. Therefore, the IVs we have selected are both exogenous and valid.

Previous studies often use school-averaged variables as IVs, which can lead to correlations with unobservable variables within schools, thereby compromising exogeneity. However, incorporating school fixed-effects to account for unobservable variables within schools may render the use of school-averaged IVs infeasible. This is due to the collinearity between these variables and fixed-effects, making them essentially a linear combination of fixed-effects and thus unable to be used simultaneously. Our IVs avoid such problem since peers’ fathers’ behaviors various between each student and are less likely to be correlated with unobserved factors of schools.

In addition, two students may become friends because they displayed similar behaviors before becoming friends, that is, their behavior is not the result of the influence of a good friend. Therefore, there may be a self-selection problem in drinking alcohol. To mitigate this, we added the question “Why is he/she your best friend?” to the questionnaire. According to the students’ answers, 71% chose to become friends with their peers because of their “good personality,” while few chose friends who had similar drinking habits. Therefore, we were able to ignore the endogeneity of self-selection.

Specifically, we estimate the following equations:


(2) $$\begin{array}{l} \mathrm{drin}k_{\mathrm{peer},i}=\alpha_{0}+\alpha_{1}\mathrm{Fdrin}k_{\mathrm{peer},i}+\alpha_{2}\mathrm{awar}e_{\mathrm{peer},i}\\ \;+\alpha_{3}M+\alpha_{4}N+\alpha_{5}\delta_{i}+\nu_{i} \end{array}$$

(3) $$\mathrm{drin}k_{i}=\beta_{0}+\beta_{1}\tilde{\mathrm{drin}k_{\mathrm{peer},i}}+\beta_{2}M_{i}+\beta_{3}N_{i}+\beta_{4}\delta_{i}+\varepsilon_{i}$$

This is called two stage least square (2SLS) method in the econometric terminology. We used peers’ fathers’ drinking behaviors and peers’ perceptions regarding harm from drinking as IVs, and conducted an overidentification test in equation (2). Here, $\mathrm{drin}k_{\mathrm{peer}}$ indicates whether peers drink alcohol and $\mathrm{Fdrin}k_{\mathrm{peer}}$ indicates whether the peers’ fathers drink alcohol. Peers’ perceptions of the dangers of alcohol consumption are represented by $\mathrm{awar}e_{\mathrm{peer}}$. In equations (3), Mi represents a series of characteristic variables at the individual level of students, such as gender, age, school attendance, number of siblings and other indicators. *N_i_* represents a series of characteristic variables at the family level, such as parents’ ages, parents’ educational year, and household durable assets.

With data and methods prepared, we use STATA 17 software to estimate all the parameters that we are interested in.

## 3 Results

### 3.1 OLS and IV estimation

Based on the OLS estimation, this study investigated the relationship between peer drinking and adolescent drinking behavior. In Table 2, the dependent variable was adolescent drinking. Columns (2) and (3) are the stepwise regression results after adding students’ individual characteristics and family characteristics to the OLS model. The OLS estimation results show that adolescents who had peers who drank alcohol were 11.1% more likely to drink alcohol than those who had peers who did not. After adding individual characteristics and family characteristics into the model, the drinking probability of adolescents who had peers who drank alcohol was increased by 7.1% points in comparison to those who had peers who did not, and its promoting effect was still significant.

**Table 2:** Effects of peer drinking on adolescent drinking behavior.

Variables	(1)	(2)	(3)
	OLS	OLS	OLS
Drink_peer_	0.111^***^	0.071^***^	0.071^***^
	(0.013)	(0.012)	(0.012)
Aware		−0.220^***^	−0.215^***^
		(0.017)	(0.017)
Boy		0.139^***^	0.131^***^
		(0.011)	(0.011)
Age		−0.000	0.000
		(0.001)	(0.001)
Preschool		−0.020	−0.028
		(0.024)	(0.024)
Number of siblings			0.023^**^
			(0.010)
Local dummy			−0.487^***^
			(0.010)
Father’s age			0.036^***^
			(0.005)
Mother’s age			−0.222^***^
			(0.006)
Father’s education			−0.001
			(0.002)
Mother’s education			−0.004^**^
			(0.002)
Father drinks			0.066^***^
			(0.014)
Asset			0.014^***^
			(0.004)
Constant	0.337^***^	0.433^***^	7.451^***^
	(0.005)	(0.083)	(0.204)
School fixed effects	Yes	Yes	Yes
R-squared	0.021	0.103	0.150
Observations	10,772	10,772	10,772

OLS means ordinary least square. The value of the robust standard errors is reported in parentheses. The definitions for each of the variables are available in Table 1. ^⁎⁎⁎^Indicate significance level of 1%. ^⁎⁎^Indicate significance level of 5%.

In terms of individual control variables, male student drinking probability was 13.1% points higher than that of female student. Since the samples were students of third- and fourth-years, there was a small age gap between the sample students, so the students’ ages (months) did not appear to influence their drinking behaviors. The number of siblings had appeared to have a significant positive impact on their drinking. Siblings influence young children’s cognitive skills directly or indirectly (48). Having one additional sibling increased the probability of adolescent drinking by 2.3% points. One possible reason for this is that adolescents with more siblings have less parental care and are more susceptible to peer drinking behavior. The drinking probability of rural adolescents was found to be 48.7% points lower than that of migrant adolescents. One possible reason for this is that migrant adolescents have more opportunities to drink than rural adolescents. Students’ fathers’ ages appeared to have a significant positive impact on their children’s drinking; with each additional year of age, the probability of their child’s drinking increased by 3.6% points. Mothers’ ages, on the other hand, had a significant negative impact on their children’s drinking, with every additional year reducing the probability of their child’s drinking by 22.2% points. Students’ fathers’ years of education had no statistically significant impact on their children’s drinking behaviors, while mothers’ years of education had a significant negative impact. Increasing, mothers’ years of education by 1 year reduced the probability of their children’s drinking by 0.4% points. Adolescents whose fathers drank alcohol had a 6.6% points higher probability of drinking than those whose fathers did not drink. The above results show that parents have a significant positive relationship with adolescent drinking behavior. Household durable assets can provide a suitable environment for adolescents to drink, which are positively correlated with teenage drinking behavior.

The endogeneity problems are as follows: (1) There is a mutual influential relationship between adolescent peer drinking and control variables. That is, adolescents often spend time with their peers, and the drinking behaviors of their peers often affect the individual characteristics of adolescents, such as views regarding alcohol. (2) There may be a reverse causal relationship between adolescent drinking and peer drinking. In other words, teens who drink alcohol tend to form peer relationships more easily. (3) Although the model setting and variable selection have been considered comprehensively in this paper, there may still be missing variables and measurement errors, resulting in estimation bias.

In this paper, the IV method was used to correct possible endogeneity problems. As shown in Table 3, column (1) is the regression result using the IVs. Firstly, in order to test the problem of under-identification, the estimated values of the LM (Lagrange multiplier) statistics in the regression models of columns (1) were calculated to be 494.949 (*p* = 0.000), indicating that there was no under-identification question. Secondly, in order to test whether IVs are weak, we calculated the Cragg-Donald Wald F statistic, which was much higher than the critical value of rejecting the weak IV hypothesis at the 10% statistical level, strongly rejecting the null hypothesis of “IV redundancy.” Thirdly, through the Overidentification test of all Instruments, the Hansen J statistic was 0.957, which strongly rejected the hypothesis of endogenous instrumental variables. Therefore, the selection of IVs in this paper had strong explanatory power regarding whether the sample students drank alcohol, and the selection of IVs was deemed appropriate.

**Table 3:** Instrumental variable analysis of peer effects.

Variables	(1)	(2)	(3)
	2SLS	2SLS	2SLS
	All the samples	Peer fathers who worked outside	Peer fathers who worked locally
Drink_peer_	0.105^***^	0.126^**^	0.097^*^
	(0.040)	(0.062)	(0.053)
Aware	−0.214^***^	−0.201^***^	−0.223^***^
	(0.010)	(0.015)	(0.013)
Boy	0.126^***^	0.137^***^	0.115^***^
	(0.010)	(0.016)	(0.014)
Age	−0.000	0.001^*^	−0.001^*^
	(0.000)	(0.001)	(0.000)
Preschool	−0.028^*^	−0.023	−0.033
	(0.017)	(0.027)	(0.021)
Number of siblings	0.023^***^	0.032^***^	0.015^*^
	(0.007)	(0.010)	(0.009)
Local dummy	−0.477^***^	−0.355	−0.401
	(0.148)	(0.230)	(0.252)
Father’s age	0.032	0.066	−0.001
	(0.046)	(0.066)	(0.064)
Mother’s age	−0.214^***^	−0.226^***^	−0.112
	(0.052)	(0.062)	(0.112)
Father’s education	−0.000	−0.003	0.003
	(0.002)	(0.003)	(0.002)
Mother’s education	−0.004^***^	0.001	−0.008^***^
	(0.001)	(0.002)	(0.002)
Father drinks	0.066^***^	0.068^***^	0.067^***^
	(0.009)	(0.014)	(0.011)
Asset	0.014^***^	0.018^***^	0.010^***^
	(0.003)	(0.005)	(0.004)
Constant	7.326^***^	0.126^**^	4.848
	(2.070)	(0.062)	(4.203)
School fixed effects	Yes	Yes	Yes
Observations	10,772	4,726	6,046
Cragg-Donald Wald F statistic	306.383	126.378	177.466
Hansen J statistic	0.957	1.458	4.994
Permutation test (P-val)		0.316	

2SLS means two stages least square method. The values of the robust standard errors are reported in parentheses. The definitions for each of the variables are available in Table 1. ^⁎⁎⁎^Indicate significance level of 1%. ^⁎⁎^Indicate significance level of 5%. ^*^Indicate significance level of 10%.

In Table 3, the regression results in column (1) show that peer drinking had a positive influence on students’ drinking with peers’ perceptions of drinking harm and peers’ fathers’ drinking behaviors as IVs. Adolescents who had peers who drank alcohol were 10.5% points more likely to drink than those who had peers who did not. The above regression results show that peer drinking increased the probability of students drinking. At the same time, the regression results using IVs also showed that family and regional characteristics (e.g., father’s drinking behavior and parents’ years of education) significantly affect students’ drinking behaviors.

In order to test the robustness of the results, the sample was divided into peer fathers who worked outside the home (columns 2 of Table 3) and peer fathers who worked locally (columns 3 of Table 3). In the group of peer fathers who worked outside, the drinking probability of adolescents who had peers who drank alcohol was increased by 12.6% points in comparison to those who had peers who did not. In the other group, the drinking probability of adolescents who had peers who drank alcohol was increased by 9.7% points in comparison to those who had peers who did not. The results showed that the peer effect still had a positive influence on adolescent drinking behavior in the two group regressions, indicating the robustness of the results. A permutation test was used to test the difference in the coefficients between the groups after grouping regression. The empirical *p* value of this difference was 0.316 under 2SLS model, which is not statistically significant, indicating that there is no significant difference between the regression coefficients of the two groups.

### 3.2 Heterogeneity analysis results

Based on existing literature research, there are individual, regional (49), and family differences (50) in adolescent drinking behavior. In order to identify these differences, we analyzed the heterogeneity for three aspects: migrant or rural, left-behind, or non-left behind, and parents’ migrant work.

To overcome potential endogeneity problems, we used the 2SLS (two stage least square) model for regression after the OLS regression (Table 4). Columns (1) and (2) are the OLS and 2SLS models (with peer drinking and peer perception of harm from drinking as IVs) respectively. In the regression of the 2SLS model, the test results of LM and Cragg Donald Wald F statistics show that there is no insufficient identification or weak IVs, indicating that the selection of instrumental variables is still appropriate. Therefore, the grouped regression coefficients reported below refer to the results of the 2SLS model.

**Table 4:** Results of heterogeneity analysis of peer effect.

Variables	(1)	(2)
	OLS	2SLS
	**Migrant (*N* = 4,659)**	
Drink_peer_	0.045^***^	0.100^*^
	(0.015)	(0.059)
R-squared	0.137	0.135
Control variables	Yes	Yes
School fixed effects	Yes	Yes
Cragg-Donald Wald F statistic		140.243
Overidentification test (P-val)		0.022
	**Rural (*N* = 6,113)**	
Drink_peer_	0.089^***^	0.114^**^
	(0.013)	(0.055)
R-squared	0.162	0.161
Control variables	Yes	Yes
School fixed effects	Yes	Yes
Cragg-Donald Wald F statistic		166.031
Overidentification test (P-val)		0.570
Permutation test(P-val)		0.249
	**Non-left behind children (*N* = 1732)**	
Drink_peer_	0.067^***^	0.084
	(0.024)	(0.087)
R-squared	0.219	0.219
Control variables	Yes	Yes
School fixed effects	Yes	Yes
Cragg-Donald Wald F statistic		59.987
Overidentification test (P-val)		0.463
	**Left-behind children (*N* = 4,381)**	
Drink_peer_	0.098^***^	0.130^*^
	(0.015)	(0.067)
R-squared	0.171	0.118
Control variables	Yes	Yes
School fixed effects	Yes	Yes
Cragg-Donald Wald F statistic		109.553
Overidentification test (P-val)		0.697
Permutation test(P-val)		0.232
	**Neither parent is at home (*N* = 2,901)**	
Drink_peer_	0.122^***^	0.223^***^
	(0.019)	(0.086)
R-squared	0.193	0.106
Control variables	Yes	Yes
School fixed effects	Yes	Yes
Cragg-Donald Wald F statistic		66.153
Overidentification test (P-val)		0.653
	**Father at home (*N* = 312)**	
Drink_peer_	0.137^**^	0.557^**^
	(0.061)	(0.258)
R-squared	0.477	0.001
Control variables	Yes	Yes
School fixed effects	Yes	Yes
Cragg-Donald Wald F statistic		5.340
Overidentification test (P-val)		0.320
Permutation test(P-val)		0.151

OLS means ordinary least square. 2SLS means two stages least square. The values of the robust standard errors are reported in parentheses. The definitions for each of the variables are available in Table 1. ^⁎⁎⁎^Indicate significance level of 1%. ^⁎⁎^Indicate significance level of 5%. ^*^Indicate significance level of 10%.

Firstly, we divided the sample into migrant and rural adolescents to investigate differences in regards to the residence of rural students. Overall, the numbers of migrant and rural adolescents drinking alcohol were similar, with the proportion of rural and migrant adolescents drinking being 28.17 and 24.40%, respectively. The results of a migrant-rural heterogeneity analysis show that peer drinking can significantly increase the drinking behavior of migrant and rural adolescents. In the rural students, adolescents who had peers who drank alcohol has a 11.4% point higher probability than those who had peers who did not, and its thrust is significantly higher than that of migrant students. Whether the coefficient difference between the two sub-samples is significant still needs to be tested. A permutation test was used to test the coefficient difference between the groups after the grouping regression. The test results show that there was no significant difference in the peer effect coefficient of drinking among migrant and rural adolescents.

Secondly, in order to analyze the peer effect of drinking behavior of rural adolescents, the students were divided into two groups: non-left behind children (NLBC) and left behind children (LBC). We define left-behind children as children who have at least one of their parents working outside the home. The regression results show that there was no peer effect in NLBC group and a significant peer effect in LBC group. The permutation test was used to test the coefficient difference between the groups after grouping regression. The test results show that the coefficient difference passed the significance level test and there is a significant difference. The results further show that for rural adolescents, the drinking behavior of NLBCs is not affected by peer drinking because they receive care from their parents. On the other hand, the lack of parental care provided to LBCs leads to the significant peer effect on their drinking. Among the LBC, adolescents who had peers who drank alcohol were 13.0% points more likely to drink than those who had peers who did not.

Thirdly, we focused on the difference in peer effects between the LBCs group with all parents out and the LBCs group with fathers at home. In order to further analyze the peer effect of parental care on adolescent drinking behavior, students were divided into two groups: neither parent is at home (NH) and father at home (FH). In the NH and FH groups, peer drinking significantly affected adolescent drinking behavior, and the peer effect coefficient in FH group was more than that in NH group. Without parental care, adolescents are more likely to be influenced by peer drinking, and adolescents who had peers who drank alcohol has a 22.3% point higher probability than those who had peers who did not. However, in the FH group, although the weak instrumental variable test fails due to too few samples, the result is still significant.

## 4 Discussion

This paper aimed to estimate the peer effect of drinking on adolescent drinking behavior in China, using the drinking behaviors of peers’ fathers and their beliefs about the health risks of alcohol as instrumental variables. The main findings of this paper are as follows:

Firstly, peer drinking significantly influences adolescent drinking behavior, with adolescents who have peers who drink alcohol being 10.5% points (c.f. the coefficient of Drink_peer_ in Table 3, which is 0.105 and is both economically and statistically significant) more likely to engage in drinking compared to those without such peers. This effect is robust to different specifications and subsamples.

Secondly, the peer effect differs significantly between migrant and rural adolescents, left-behind and non-left-behind adolescents, and adolescents with different parental care situations. The peer effect is stronger for rural adolescents, left-behind adolescents, and adolescents whose parents work outside the home.

Finally, parental care plays a significant role in the degree of peer effect, with the absence of parental care being a key factor in the presence of the peer effect. Parental characteristics, such as age, education, and drinking behavior, also have significant impacts on adolescent drinking behavior.

These findings contribute to the existing literature on adolescent drinking behavior in several ways. First, this paper provides novel and rigorous evidence on the peer effect of drinking on adolescent drinking behavior in China, a country with a large and diverse population of adolescents and a high prevalence of underage drinking. Previous studies on this topic either have only identified the causal effect of peer drinking (51, 52), or have often relied on weak or questionable instrumental variables, such as school-average variables, to address the endogeneity problem. The consequence of omitting the dual-causality problem can lead to biased estimation. For instance, a study estimating the peer effect which overlooks such endogeneity problem yields a 22% effect of alcohol drinking probability increase by peer drinking behavior (53), which is nearly 2 times greater than our result:10.5%. This paper uses more powerful and valid instrumental variables, based on the detailed information of household background in the dataset, to overcome the endogeneity issue and obtain consistent and unbiased estimates of the peer effect.

Second, this paper highlights the importance of parental care in moderating the peer effect and influencing adolescent drinking behavior. Previous studies highlighted the importance of both parental monitoring and peer influence in adolescent alcohol use, with parental monitoring having an indirect effect on drinking behavior through its influence on peer use and tolerance (54). Another research found that adolescents with binge-drinking parents were more likely to increase their own drinking regardless of the level of peer drinking (55). Our study claim that parental care is a crucial factor that affects the development and well-being of adolescents, especially in the context of rapid urbanization and industrialization in China, which have led to large-scale migration and separation of families. This paper shows that parental care can reduce the susceptibility of adolescents to peer influence and protect them from engaging in harmful drinking behavior. Parental characteristics, such as age, education, and drinking behavior, also have significant impacts on adolescent drinking behavior, suggesting that parents can serve as role models and sources of information and guidance for their children.

This paper also has some limitations, biases, or imprecisions that should be acknowledged and addressed in future research. First, this paper relies on self-reported data on adolescent and peer drinking behavior, which may be subject to measurement errors, reporting biases, or social desirability biases. For example, adolescents may underreport or overreport their own or their peers’ drinking behavior due to fear of punishment, peer pressure, or impression management. Future research could use more objective and reliable measures of adolescent and peer drinking behavior, such as biomarkers, administrative records, or direct observations.

Second, this paper uses the best friend reported by the student as the proxy for the peer group, which may not capture the full range and diversity of peer influences that adolescents are exposed to. For example, adolescents may have multiple or changing best friends, or they may be influenced by other peers who are not their best friends, such as classmates, neighbors, or online friends. Future research could use more comprehensive and dynamic measures of peer groups, such as network data, social media data, or longitudinal data, to better understand the structure and evolution of peer relationships and influences.

Third, this paper focuses on the peer effect of drinking on adolescent drinking behavior, but does not examine the potential spillover effects of peer drinking on other outcomes, such as academic performance, mental health, or risky behaviors. Peer drinking may have positive or negative effects on these outcomes, depending on the nature and context of peer interactions and influences. For example, peer drinking may foster social bonding and emotional support, or it may impair cognitive functioning and increase impulsivity and aggression. Future research could explore the broader and longer-term consequences of peer drinking for adolescents and their families, communities, and society. The mechanisms of peer effect, such as social norms, peer pressure, parental monitoring, or parental communication can be further investigated.

## 5 Conclusion

This paper finds a significant peer effect on adolescent drinking behavior. Peer drinking significantly promotes adolescent drinking behavior. And the absence of parental care is increasing the peer effect.

As schools are a large part of society, especially for adolescents, schools should strengthen education and create a good environment for their students. Our results show that adolescent drinking is significantly influenced by their peers. School culture can directly affect peers. Therefore, the role of schools in educating people should be strengthened. Schools should hold moral education classes regularly to inculcate good drinking habits among adolescents. Regarding to the importance of parental care, among rural students, more attention should be paid to left-behind children, especially families in which both parents go out to work.

Schools should pay attention to left-behind children in rural areas, and establish a linkage mechanism between guardians and schools. Our results show that left-behind children in rural areas are more susceptible to peer behavior due to lack of parental care. Schools shall organize regular parent-teacher meetings to provide timely feedback to parents on the performance of adolescents in school. For parents who go out to work, schools should communicate regularly with parents through WeChat groups and phone calls. In terms of guardianship of left-behind children, schools, therefore, should collect feedback in a timely manner and establish a communication mechanism with guardians.

For the future studies, we mainly recommend researchers follow the following directions:

Firstly, expand the sample size and scope. The paper uses data from only four provinces in China, which may not be representative of the whole country. A larger and more diverse sample could increase the external validity and generalizability of the findings.

Secondly, use longitudinal data and dynamic models. The paper relies on cross-sectional data and static models, which may not capture the temporal and causal relationships between peer drinking, parental care, and adolescent drinking behavior. Longitudinal data and dynamic models could allow for tracking the changes and impacts of these variables over time.

Finally, explore the mechanisms and channels of peer and parental influences. The paper does not examine the underlying mechanisms and channels through which peer and parental influences affect adolescent drinking behavior, such as social norms, peer pressure, parental monitoring, or parental communication. Understanding these mechanisms and channels could help design more effective interventions and policies to prevent and reduce underage drinking.

## Data availability statement

The raw data supporting the conclusions of this article will be made available by the authors, without undue reservation.

## Ethics statement

The studies involving humans were approved by China Agricultural University Institutional Review Board. The studies were conducted in accordance with the local legislation and institutional requirements. Written informed consent for participation in this study was provided by the participants’ legal guardians/next of kin.

## Author contributions

ML: Conceptualization, Data curation, Formal analysis, Methodology, Software, Visualization, Writing – original draft, Writing – review & editing. W-QZ: Conceptualization, Data curation, Formal analysis, Methodology, Software, Writing – original draft, Writing – review & editing. Q-RZ: Data curation, Investigation, Methodology, Resources, Writing – original draft. YW: Data curation, Investigation, Methodology, Resources, Writing – original draft. S-GL: Project administration, Writing – review & editing, Funding acquisition.
